# Effective Utilization of Waste Red Mud for High Performance Supercapacitor Electrodes

**DOI:** 10.1002/gch2.201800066

**Published:** 2018-10-25

**Authors:** Gourav Bhattacharya, Sam Jeffery Fishlock, Joy Sankar Roy, Anurag Pritam, Debosmita Banerjee, Sujit Deshmukh, Subhasis Ghosh, James A. McLaughlin, Susanta Sinha Roy

**Affiliations:** ^1^ Nanotechnology and Integrated Bioengineering Centre University of Ulster Jordanstown Campus Newtownabbey BT37 0QB Northern Ireland UK; ^2^ Department of Physics School of Natural Sciences Shiv Nadar University Gautam Buddha Nagar 201314 Uttar Pradesh India; ^3^ School of Physical Sciences Jawaharlal Nehru University New Delhi 110067 India

**Keywords:** long‐term cyclic stability, red mud, supercapacitors, waste management

## Abstract

In recent years, metal oxide‐based, inexpensive, stable electrodes are being explored as a potent source of high performance, sustainable supercapacitors. Here, the employment of industrial waste red mud as a pseudocapacitive electrode material is reported. Mechanical milling is used to produce uniform red mud nanoparticles, which are rich in hematite (Fe_2_O_3_), and lower amounts of other metal oxides. A comprehensive supercapacitive study of the electrode is presented as a function of ball‐milling time up to 15 h. Ten‐hour ball‐milled samples exhibit the highest pseudocapacitive behavior with a specific capacitance value of ≈317 F g^−1^, at a scan rate of 10 mV s^−1^ in 6 m aqueous potassium hydroxide electrolyte solution. The modified electrode shows an extraordinary retention of ≈97% after 5000 cycles. A detailed quantitative electrochemical analysis is carried out to understand the charge storage mechanism at the electrode–electrolyte interface. The formation of uniform nanoparticles and increased electrode stability are correlated with the high performance. This work presents two significant benefits for the environment; in energy storage, it shows the production of a stable and efficient supercapacitor electrode, and in waste management with new applications for the treatment of red mud.

## 1 Introduction

The exploitation of nonconventional renewable energy sources is of significant worldwide interest due to the increasing global demand for energy, the rapid exhaustion of fossil fuels and other nonrenewable energy resources, and environmental concerns such as global warming and climate change.^1^ Among several energy storage devices, supercapacitors, owing to their high power density, high specific capacitance, and superior cyclic stability, have a huge potential as portable power storage units, and can be used efficiently in many areas such as electric vehicles and consumer electronics.^2^, ^3^, ^4^, ^5^

The charge storage mechanism in a supercapacitor is governed either by electrical double layer capacitance (EDLC)^6^, ^7^ or by pseudocapacitance.^8^, ^9^ In EDLC, the charge is stored electrostatically through reversible adsorption of electrolytes onto a high surface area and electrochemically stable electrode. The phenomenon is related to a potential‐dependent accumulation of charges at the electrode–electrolyte interface.^10^, ^11^ In pseudocapacitance, the capacitance is faradic in origin^12^ whereby an ultrafast redox reaction takes place at or near the electrode and a faradic charge is passed as a function of electrode potential.^13^, ^14^ Carbon‐based materials such as activated carbon,^15^ carbon nanotubes,^16^ graphene,^17^, ^18^ and reduced graphene oxide^19^, ^20^ have been explored as EDLC materials whilst conducting polymers^21^, ^22^ and transition metal oxides^23^, ^24^ have been utilized as pseudocapacitive supercapacitor electrodes.

One of the most important factors for supercapacitor performance is the electrode material,^1^ and researchers across the globe are seeking inexpensive, stable, and high‐performance electrode materials.^25^, ^26^ In the modern era of industrialization, a huge amount of different forms of organic and inorganic waste byproducts have been accumulating in the environment, resulting in air–water–soil pollution and an overall degradation of our ecosystems and quality of life.^27^, ^28^, ^29^ Over the last decade, there is a worldwide drive toward waste management and researchers are utilizing the byproducts in diverse directions for the betterment of the society.^30^, ^31^ For example, different forms of agro‐industrial organic waste have been effectively utilized for supercapacitor electrodes where biomass is converted to activated carbon.^32^ A myriad of different agricultural waste types such as cassava peel waste,^33^ coffee beans,^34^ sugarcane bagasse,^35^ rice husk,^36^ sunflower seed shell,^37^ coffee endocarp,^38^ apricot shell,^39^ rubber wood sawdust,^40^ oil palm empty fruit bunch,^41^ argan seed shell,^42^ bamboo species,^43^ and oily sludge^44^ are used as starting precursors for the porous activated carbon for EDLC. Chang et al. utilized waste filter papers as precursors to synthesize the activated carbon electrodes.^45^ There are a few recent reports on the utilization of inorganic wastes to produce activated carbon materials. Zhi et al. used waste tires.^46^ Konikkara et al. synthesized activated carbon from solid leather waste and used as an EDLC.^47^ Though wastes have been utilized in EDLC supercapacitors, there is hardly any report on the waste‐derived noncarbonic pseudocapacitor electrode. Recently, Fu and Grant produced a supercapacitor electrode based on industrial mill scale waste (iron oxide enriched) which exhibits a promising storage capability.^48^

Red mud (RM) is an industrial waste which is generated during the bauxite ore processing.^49^ Around 120 million tons of red mud and other bauxite residues are produced yearly^50^ and there have been many large‐scale environmental disasters involving red mud, recently in Hungary (2010)^51^ where ten persons were directly killed in a red mud flood, over one hundred more injured, and a large area of land and river was environmentally decimated. Thus, red mud is an abundant material which has been repeatedly shown to cause environmental horrors. This byproduct is alkaline in nature^52^ and contains a rich mixture of metal oxides.^53^ This waste is very common in India and after activating the red mud, researchers have to date mainly utilized it in wastewater treatment systems such as dye degradation,^54^ arsenic, and other heavy metal adsorption processes.^55^, ^56^ Though it comprises more than 50% hematite (Fe_2_O_3_) which has been deployed extensively in supercapacitor electrodes, surprisingly, the potential of red mud as a pseudocapacitive material has not been explored yet.

In this report, the potential of hazardous red mud as a pseudocapacitor material has been explored. The as‐received red mud from the industry was mechanically milled using a ball‐milling technique to produce uniform red mud nanoparticles. The milling time was varied as a function of particle size and electrode stability. The evaluation of electrochemical properties of mechanically activated waste nanoparticles exhibited impressive supercapacitor behavior with a remarkable long‐term stability. Furthermore, an in‐depth electrochemical analysis has been undertaken to understand the origin of the storage mechanism, and hence we have established the feasibility of red mud as a promising, inexpensive electrode material.

## 2 Results and Discussion

### 2.1 Morphology Analysis of RM‐0

**Figure 1:**
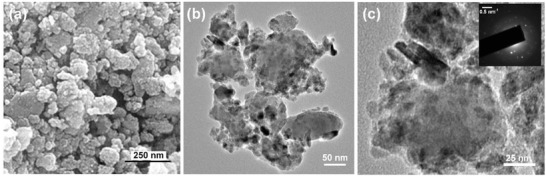
a) FESEM image and b,c) TEM images of RM‐0. Inset of (c) exhibits the corresponding SAED pattern of RM‐0.

**Figure 2:**
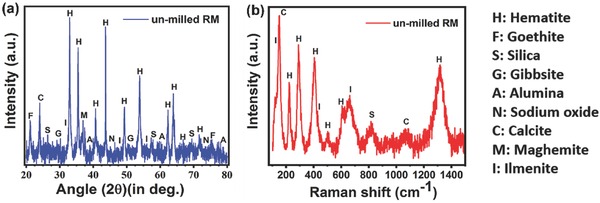
a) XRD pattern and b) Raman spectroscopy measurement for RM‐0.

The as‐obtained RM (we term this RM‐0, denoting 0 h ball milling, and subsequently, the RM‐3 sample has been milled for 3 h etc.) powders were at first dehydrated in an oven for 1 h at 383 K and then ground to obtain moisture and chunk‐free microparticles. The chemical components were analyzed using WDXRF analysis and are represented in **Table** **1**. From the table, it is quite evident that RM consists mainly of oxides of iron [hematite and maghemite (Fe_2_O_3_) (≈55%)] and alumina (Al_2_O_3_). It also includes quartz (SiO_2_), complex oxides of titanium, and trace amount of various other metal oxides (magnesium, manganese, sodium, etc.). Field‐effect scanning electron microscopy (FESEM) imaging was used to give a direct morphological visualization of this red mud powder and is shown in **Figure** **1**a. FESEM shows that RM‐0 red mud particles have no uniform shape, size, and particle distribution. Representative transmission electron microscopy (TEM) images of RM‐0 is shown in Figure 1b,c. A large variation in particle size and shape distribution can be observed from the TEM images of RM‐0. The inset of Figure 1c exhibits the selected area electron diffraction (SAED) pattern of RM‐0, where different polycrystalline phases of several metal oxides can be observed. An energy dispersive X‐ray (EDX)‐TEM study of RM‐0 is presented in Figure S1 in the Supporting Information. The presence of iron, aluminum, silicon, sodium, calcium, titanium, and manganese is confirmed from the EDX spectra. To further confirm the crystalline phases, wide‐angle X‐ray diffraction (XRD) analysis was carried out and is represented in **Figure** **2**a. The XRD patterns are similar to the previous reports^57^, ^58^ and show the presence of crystalline hematite, maghemite, goethite, alumina, ilmenite, sodium oxide, gibbsite, calcite, and silica.^58^, ^59^

**Table 1:** Wavelength‐dispersive X‐ray fluorescence (WDXRF) data of RM‐0 samples

Constituents	% w/w
Iron oxide	≈55
Aluminum oxide	≈15
Silica	≈7
Sodium oxide	≈5
Titanium oxide	≈4
Calcium oxide	≈3
Others	≈11

Raman spectroscopy was also carried out to probe different vibrational modes of the metal oxides present in the red mud and represented in Figure 2b. The main peaks were attributed to the following modes: Fe_2_O_3_ hematite: *E*_g_ mode at 291 and 404 cm^−1^, A_1g_ at 223 and 502 cm^−1^;^60^ ilmenite FeTiO_3_: E_g_^5^ mode at 146, and A_G_^1^ at 662 cm^−1^;^61^ CaCO_3_ calcite: *E*_g_ mode at 152 cm^−1^.^62^

### 2.2 Electrochemical Characterization

#### 2.2.1 Cyclic Voltammetry (CV) Study

**Figure 3:**
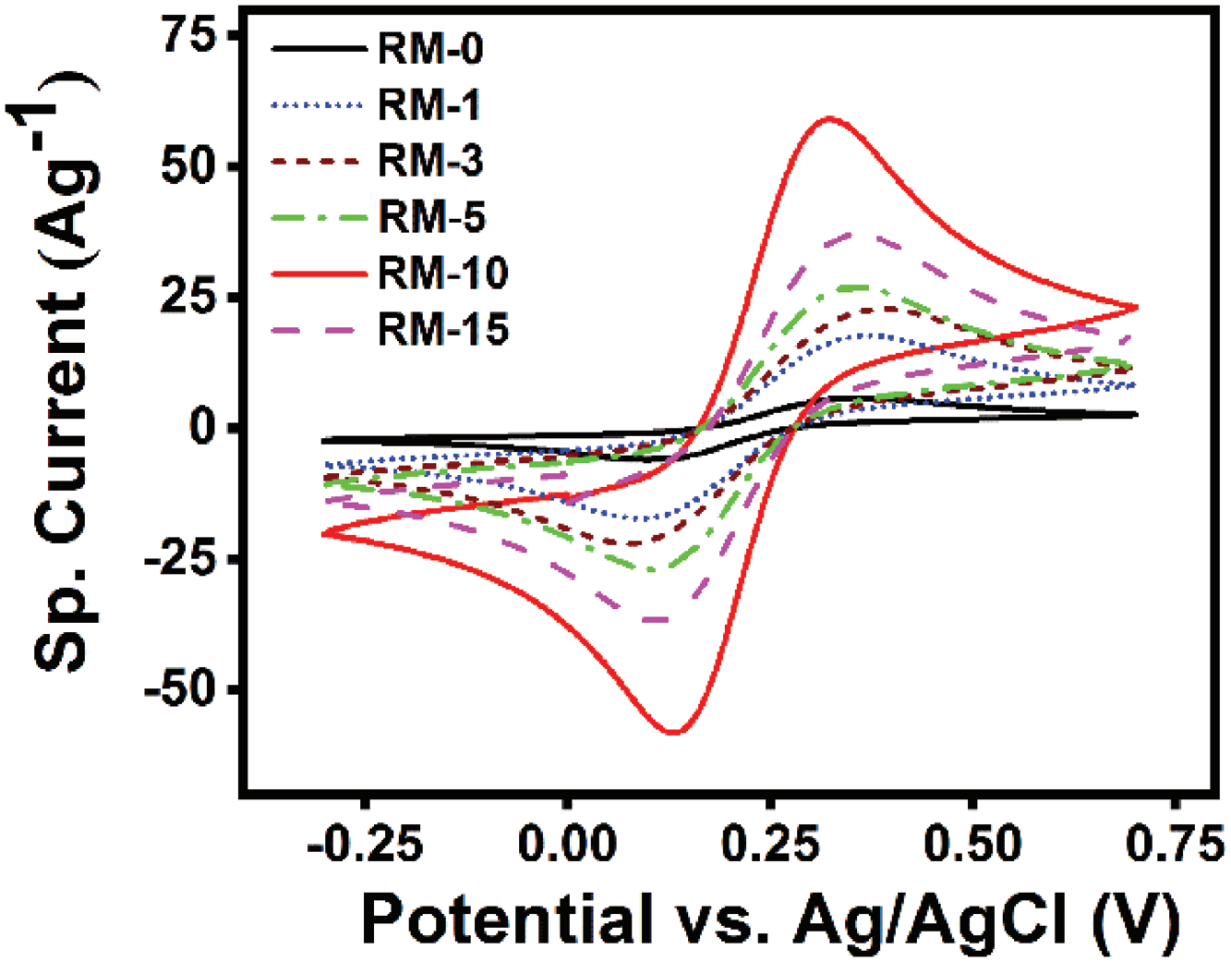
Cyclic voltammogram of RM‐0, RM‐1, RM‐3, RM‐5, RM‐10, and RM‐15 modified GCE in the solution of 0.1 m KCl containing 5 × 10^−3^ m [Fe(CN)_6_]^3−/4−^ with a scan rate 50 mV s^−1^.

In order to find out the effect of ball milling on the red mud samples, cyclic voltammetry was carried out on a three‐electrode system with red mud modified glassy carbon electrode (GCE) as the working electrode, platinum counter electrode, and Ag/AgCl as a reference electrode. **Figure** **3** represents CV scans of all red mud samples. CV scans were executed using a 0.1 m KCl aqueous solution containing 5 × 10^−3^ m Fe(CN)^3−/4−^ redox couple within a scan range −0.30 to +0.70 V with a scan rate of 50 mV s^−1^. For the RM‐0 powder, a set of redox peaks are observed around ≈0.10 V (cathodic) and ≈0.56 V (anodic) with the peak anodic and cathodic current of 5.8 and −5.9 A g^−1^, respectively. With increased milling time, the peak currents are found to enhance, with maximum peak currents of 59.1 and −58.0 A g^−1^ observed in RM‐10. There is a significant (approximately tenfold) enhancement in the peak current density which is suggestive of improved charge transfer kinetics^63^ and a better electron transfer pathway in case of RM‐10 sample. Different voltammetry parameters such as the anodic and cathodic peak potentials, separation potential, coulombic efficiency, and half‐cell potential are calculated for all samples (RM‐0 to RM‐15) as represented in **Table** **2**. The separation potential (the difference between the anodic and cathodic peak potentials) (Δ*E*_diff_) is a qualitative measurement of reversibility of redox reactions, where a smaller value indicates a better reversibility.^64^ Δ*E*_diff_ was measured to be ≈258 mV for RM‐0 sample, indicating the redox reaction is somewhat quasi‐reversible in nature. The Δ*E*_diff_ for RM‐3 sample was found to be ≈313 mV implying a poor reversibility. The value is least for the RM‐10 sample (≈195 mV) and thus the reversibility is much improved. Further milling re‐enhanced Δ*E*_diff_ (≈305 mV for RM‐15 particles).

**Table 2:** Experimental results of cyclic voltammetry measurements for RM‐0, RM‐1, RM‐3, RM‐5, RM‐10, and RM‐15 modified GCE

Sample	Anodic peak potential ($E_{a}^{0}$) [mV]	Cathodic peak potential ($E_{a}^{0}$) [mV]	Separation potential (Δ*E*_dif_) [mV]	Coulombic efficiency [η%]	Half‐cell potential $\left(E_{1/2}^{0}\right)$ [mV]
RM‐0	361	103	258	96	232
RM‐1	371	090	281	97	230
RM‐3	386	073	313	97	229
RM‐5	352	105	247	98	228
RM‐10	325	129	196	98	227
RM‐15	383	078	305	97	230

The ratio of cathodic to anodic peak current represents the coulombic efficiency (η%) of the electrode materials. η was further evaluated from the cyclic voltammetry curve and is also tabulated in the table (Table 2). The η value for the RM‐0 was found to be 96%, inferring a high charge retention of 5 × 10^−3^ m Fe(CN)^3−/4−^ redox couple in 0.1 m KCl solution. All samples exhibit efficiencies >96% with the maximum of ≈98% being obtained for the RM‐10 sample.

The mean of the cathodic and anodic peak potentials (half‐cell potential) can be used to estimate electrochemical reversibility and the cell stability of the electrode material. The half‐cell potential as a function of the milling time is shown in Table 2. From the table it is evident that there is a constant drop in the half‐cell potential with milling times up to 10 h, to a minimum of ≈227 mV. Up to 10 h of ball milling, we observed a significant enhancement in current density, and continuous decrement in half‐cell potential, and thus RM‐10 samples unveiled the best electrochemical performance. After more than 10 h of milling, the half‐cell potential interestingly increased to ≈231 mV for the RM‐15 powder, which demonstrates a slight reduction in electrode stability. This is also consistent with the previous observations: i) the current density for RM‐15 samples were lower, ii) Δ*E*_diff_ was higher, and iii) the coulombic efficiency reduced in comparison to the RM‐10 sample. In the following section, the morphological and structural changes in the red mud particles during milling will be explored and correlated to the electrochemical data.

### 2.3 Morphology Analysis of Ball‐Milled RM

**Figure 4:**
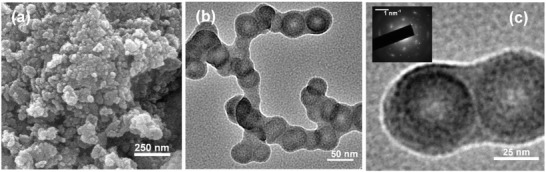
a) FESEM image and b,c) TEM images of RM‐10. Inset of (c) exhibits the corresponding SAED pattern of RM‐10.

The FESEM images for the ball‐milled samples are shown in Figure S2 in the Supporting Information. In general, from the Figure S2 in the Supporting Information, it is evident that the particle size tends to reduce with the increase in ball‐mill time. In addition to this, the particle shape changes to become more spherical in nature. The particle size tends to decrease with milling time up to 10 h and then after, further reduction is seized. The FESEM image for RM‐10 is shown in **Figure** **4**a, where a uniform particle size distribution can be observed. The TEM images for RM‐10 sample are shown in Figure 4b,c, which showed uniform distributions of spherical, crystalline RM nanoparticles of diameter 30–50 nm. The inset of Figure 4c exhibits the SAED pattern of RM‐10, where the polycrystalline phases of different metal oxides can be seen.

Particle size analysis is a semiquantitative approximation to get an estimate of the particle size distribution. In order to draw a correlation between particle size with milling time, particle size analysis was carried out and is represented in Figure S3 in the Supporting Information. From the figure, it is evident that the average particle size of RM‐0 (Particle size ≈210 ± 40 nm) reduces with ball milling up to 10 h of milling. For RM‐10 sample, the average particle size was found to be ≈45 ± 10 nm. Interestingly, the average particle size slightly increased for RM‐15 sample (diameter ≈65 ± 20 nm). It is reported earlier that longer ball‐milling process introduces grain growth (due to cold welding) and microstrains in the system and as a consequence, the particle size increased.^65^, ^66^ Thus, the possible grain growth in RM‐15 sample is responsible for the reduction in current density and enhancement in half‐cell potential. RM‐10 sample is found to offer the best electrochemical properties and thus will be explored further for supercapacitor application. For comparison purposes, the untreated RM‐0 particles have been utilized.

### 2.4 Supercapacitor Analysis

#### 2.4.1 Cyclic Voltammetry

**Figure 5:**
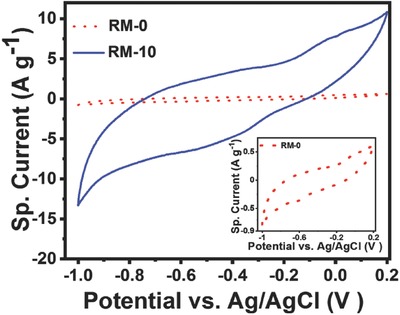
Cyclic voltammogram response of RM‐0 and RM‐10 modified GCE in 6 m KOH solution with a scan rate of 10 mV s^−1^. Inset shows the cyclic voltammogram response of RM‐0 modified GCE in similar test conditions.

**Figure 6:**
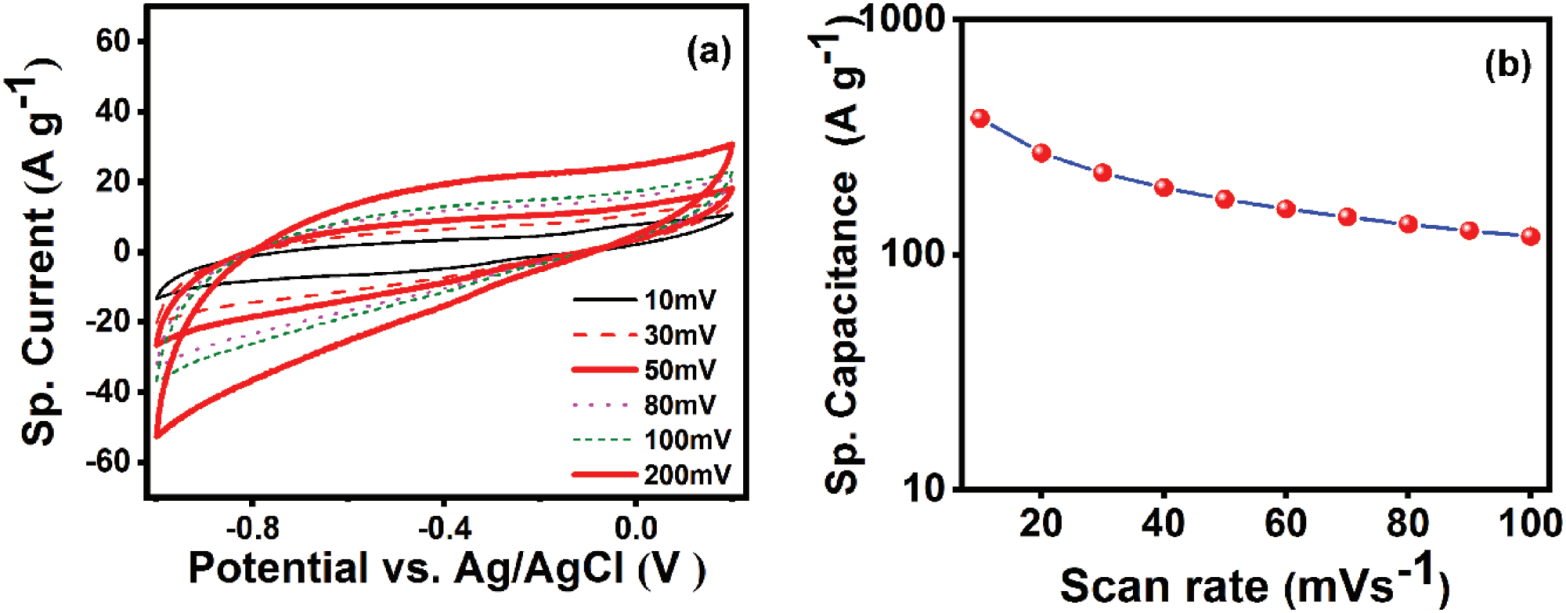
a) Cyclic voltammogram of RM‐10 sample in 6 m KOH solution in different scan rate. b) the variation of sp. capacitance with scan rate for RM‐10 in 6 m aqueous KOH solution.

**Figure** **5** represents the cyclic voltammetry graph for RM‐0 and RM‐10 particles in 6 m KOH solution at a scan rate 10 mV s^−1^ using three‐electrode assembly as mentioned earlier. There is a substantial enhancement in the sp. current density (≈16 times) and integrated area under the CV curve for RM‐10 particles compared to the untreated sample. RM‐10 shows a shallow broad reduction hump around −0.42 V which matches with the pseudocapacitance values of Fe_2_O_3._ 67 The charge storage mechanism in case of hematite is reported to be similar to that of the magnetite,^68^ where the reaction and storage mechanisms are correlated with the redox reactions accompanied by the diffusion mediated intercalation.^69^ **Figure** **6**a represents the cyclic voltammogram of RM‐10 sample at different scan rates from 10 to 200 mV s^−1^.

The specific capacitance of the RM 10 sample is evaluated from the CV curve by employing the following equation^70^, ^71^

(1) $$Cs=0.5\left[\frac{I}{v/m}\right]$$

where *C*_s_ is the sp. capacitance, *I* is the total current obtained from the CV curve, *v* is the scan rate, and *m* is the active mass of RM‐10 on the glassy carbon electrode. The multiplication factor 0.5 is originated to take care of either cathodic or anodic current.

The total current *I* can also be calculated by integrating the area of the CV curve defined as^71^, ^72^

(2) $$I=\int_{V_{i}}^{V_{f}}\frac{i\left(V\right)dV}{\left(V_{f}-V_{i}\right)}$$

where *V_i_* and *V_f_* are the lowest and highest voltage of the potential range. The specific capacitance of RM‐10 as a function of scan rate is plotted in Figure 6b. At the lowest experimental scan rate (10 mV s^−1^) a sp. capacitance of 317 F g^−1^ was obtained. At the same sweeping potential (10 mV s^−1^), a sp. capacitance value of 21 F g^−1^ was calculated for the RM‐0. The poor capacitive performance of the RM‐0 sample could be correlated with the nonuniform macro‐RM particles with poor electrical conductivity. At the highest experimental scan rate (200 mV s^−1^) a sp. capacitance of 72 F g^−1^ was achieved for RM‐10.

**Figure 7:**
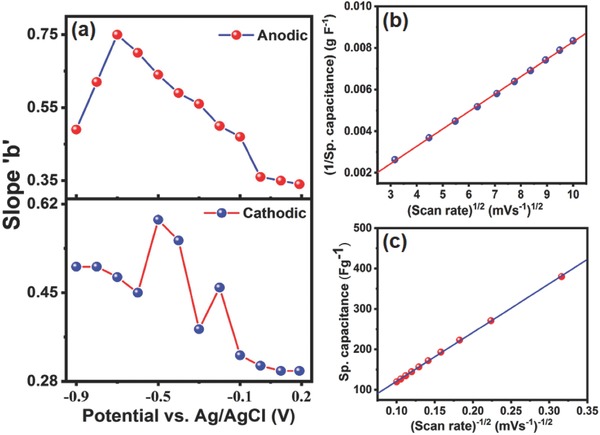
a) Dependence of slope “*b*” (derived from linear fitting of log *i* vs log *v*) as a function of cell potential and Trasatti's method for RM‐10 modified electrode for b) inverse sp. capacitance as a function of square root of scan rate and c) sp. capacitance as a function of inverse of scan rate.

The total amount of charge stored in an electrode is governed by the contributions of both capacitive and the intercalation processes. The capacitive component consists of ion adsorption/desorption reactions at the electrode/electrolyte (double layer) and very fast faradic surface redox reactions (pseudocapacitance).^73^, ^74^The appearance of these divergent energy storage mechanisms can be calculated and distinguished from the other by examining the CV scans at different scan rates according to the following power law^75^, ^76^

(3) $$i=av^{b}$$

where *i* is the scan rate dependent current, *v* is the scan rate, and *a* and *b* are the adjustable parameters. When *b* is close to 1, the current is predominantly capacitive in nature, whereas *b* values closer to 0.5 signify the current flow at any fixed potential relates to a diffusion‐controlled phenomenon.^73^ Using this technique, charge storage kinetics at each potential can be mapped. To determine *b* values, log *i* versus log *v* was plotted for different potentials and the slope of the best linear fit data provides the *b* value. As an example, the slope of the linear graph of log *i* versus log *v* was plotted for different potentials and represented in Figure S4 in the Supporting Information. The *b* values for different potentials were obtained for RM‐10 in 6 m KOH solution and the variation of *b* with applied potential is plotted in **Figure** **7**a, which suggests that there is a large variation in the *b* values at different potentials. Maximum and minimum “*b”* values of 0.75 and 0.30, respectively, were obtained which suggests that the charge storage mechanism in the RM‐10 system is a combination of both surface‐dependent capacitive charge storage and diffusion driven intercalation/deintercalation phenomena.^74^ In order to make out how the electrode structure offers such explicit distinctions and to differentiate the sp. capacitance contribution from the inner and outer surface of the electrode, Trasatti's method^74^, ^77^, ^78^ was employed.

The basic method originates from the postulation that the surface and diffusion‐controlled storage mechanisms are manifested by different kinetic models and have different responses toward the sweeping rate.^77^ When the scan rate is high, the charge is stored only at the easily accessible outer surface because of the slower diffusion of electrolyte ion to the inner surface of RM‐10. Thus, the contributions from the subsurface regions can be excluded. On the other hand, at relatively low scan rate the ions can diffuse through and the total charge stored is an additive effect of both the inner and outer surface of the RM‐10 modified glassy carbon electrode. Figure 7b exhibits a plot of 1/*C*_s_ for RM‐10 as a function of square root of sweeping rate (*v*) within 10–100 mV s^−1^. In this region, the contribution of both surface and diffusion‐driven storage mechanisms are prominent.^74^ The sp. capacitance at very slow scan rates was estimated from the intercept of the best fit linear plot with the 1/*C*_s_ axis.^77^ The total sp. capacitance of 417 F g^−1^ was obtained. In order to extract the surface‐governed capacitance, the specific capacitance (*C*_s_) graph was plotted as a function of *v* ^−1/2^ and represented in Figure 7c. Here the linear region intercepts of the plot with the *C*_s_ predicted the surface governed capacitance at higher scan rate as 76 F g^−1^. After calculation, the results predict that the 82% of the total sp. capacitance arises from the diffusion‐controlled processes and the residual capacitance of 18% is surface governed. Thus the charge storage mechanism is dominated by diffusion controlled pseudocapacitance.^13^ The presence of only pseudocapacitive metal oxides in RM and absence of any porous carbon‐based (higher surface area) material limit the surface capacitance^79^ and as the RM‐10 modified GCE electrode stores more charges in the inner surface due to the low ionic solvation radius^80^ and high ionic mobility of the electrolyte.^80^ The presence of surface adsorbed solvent molecules (water), which could enhance the accessibility of ions into electrodes, may facilitate the improved ionic diffusivity.^81^

#### 2.4.2 Galvanostatic Charging/Discharging Analysis

**Figure 8:**
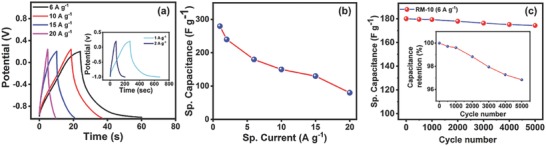
a) Galvanostatic charge/discharge curve for gravimetric capacitance of RM‐10 sample in 6 m KOH aqueous solution at different sp. current (1, 2, 6, 10, 15, and 20 A g^−1^). b) Variation of sp. capacitance with specific current for RM‐10. c) The cycling performance of RM‐10 sample in 6 m KOH solution at sp. current 6 A g^−1^ (up to 5000 cycle). Inset of (c) exhibits the percentage of capacitance retention as a function of charge/discharge cycle (up to 5000 cycles).

To determine the sp. capacitance and cyclic stability, the RM‐10 modified GCE was galvanostatically charged/discharged within the same potential range as in the CV scan (−1.0 to 0.2 V vs Ag/AgCl reference electrode), in the same 6 m KOH electrolyte. The specific capacitance values for RM‐10 nanoparticles from the galvanostatic charging/discharging analysis were evaluated from the following equation^70^

(4) $$C_{s}=-\left[\frac{i}{\left(dV\mathrm{/d}t\right)m}\right]$$

where *C*_s_ is the gravimetric sp. capacitance, *m* is the active mass of the electrode, and d*V*/d*t* is the average slope of the discharge cycle. The quasi‐symmetric charging–discharging cycles in **Figure** **8**a delineate a stable electrode performance^82^ whereas the curvature in the plot during discharge illustrates the presence of pseudocapacitance^70^, ^83^ which has already been discussed in the earlier section.

The charging/discharging plot (at a sp. current density of 6 A g^−1^) for RM‐0 and RM‐10 nanoparticles are represented in Figure S5 in the Supporting Information. The charge/discharge (CD) period in case of RM‐10 is higher than RM‐0 which indicates a higher charge storage capability.^70^ The sp. capacitance was further calculated using Equation (4). The specific capacitance for the RM‐10 sample is 180 F g^−1^, compared with 24 F g^−1^ for RM‐0. The nonuniform morphology and poor current density of RM‐0 are responsible for the poor capacitance in this sample. The ball‐milled sample (RM‐10) demonstrated much‐improved electron transfer kinetics in the CV scan: possibly due to two factors first, uniform morphology with smaller particle size, second, due to enhanced ion diffusion into the inner surfaces of the electrode.

The gravimetric capacitance was calculated from the charging–discharging graphs for different sp. current densities and represented in Figure 8a. The sp. capacitance as a function of sp. current density was plotted in Figure 8b. The maximum capacitance of 280 F g^−1^ was achieved at a current density of 1 A g^−1^, whereas at an extremely high sp. current density of 20 A g^−1^, a moderate capacitance of 80 F g^−1^ was obtained. The retention of a steady capacitance even in such ultrahigh sp. current density emphasizes the stability of the RM‐10 modified GCE and reinforces its suitability as a supercapacitor electrode material.^84^, ^85^

The areal capacitance was also calculated and the variation of areal capacitance as a function of sp. current density is represented in Figure S6 in the Supporting Information. At a low sp. current density of 1 mA cm^−2^, a sp. capacitance of 0.68 F cm^−2^ was achieved, whereas at an ultrahigh current density of 15 mA cm^−2^, a sp. capacitance of 0.03 F cm^−2^ was measured.

Long‐term cyclic stability is one of the key parameters to determine the performance of any supercapacitor material.^86^ Hence, to probe the long‐term cyclic stability, the charging/discharging measurement was carried out for 5000 cycles using a relatively high sp. current density of 6 A g^−1^. The sp. capacitance as a function of cycle number is plotted in Figure 8c. The system shows a remarkable capacitance retention and even after 5000 cycles, ≈97% capacitance was still retained (inset of Figure 8c). Particle agglomeration, material degradation, and leaching during charging/discharging are the prime causes of losses in capacitance.^87^ Here, the RM nanoparticles obtained by the ball‐milling process (RM‐10) are stable, and the morphological uniformity and the presence of other metal oxides (especially goethite) apart from iron oxide may slow down the agglomeration process.^88^, ^89^ In addition to this, there was hardly any material leaching in the electrolyte solution during charging/discharging which could also hinder the charge loss.^90^

Charging/discharging coulombic efficiency (η_cd_) is another important parameter and which could also provide significant information about long‐term stability and charge‐storage mechanism. η_cd_ is calculated using the following relation^85^, ^91^

(5) $$\eta_{\mathrm{cd}}=\frac{\mathrm{Charging}\;\mathrm{time}}{\mathrm{Discharging}\;\mathrm{time}}\times 100\%$$

The charging/discharging coulombic efficiency is calculated and η_cd_ was found to be ≈150%. Coulombic efficiencies with similar results (greater than 100%) have been reported in the literature;^91^ physically it means that the system can be charged rapidly and discharged at a relatively slow rate, which suggests RM‐10 may be utilized as a promising hybrid battery‐type supercapacitor.^91^ The variation in η_cd_ during cyclic test was also monitored and a 98% retention in η_cd_ value was achieved after 5000 cycles, which indicates an excellent reversibility of RM‐10 modified GCE during charge/discharge process.^90^

**Figure 9:**
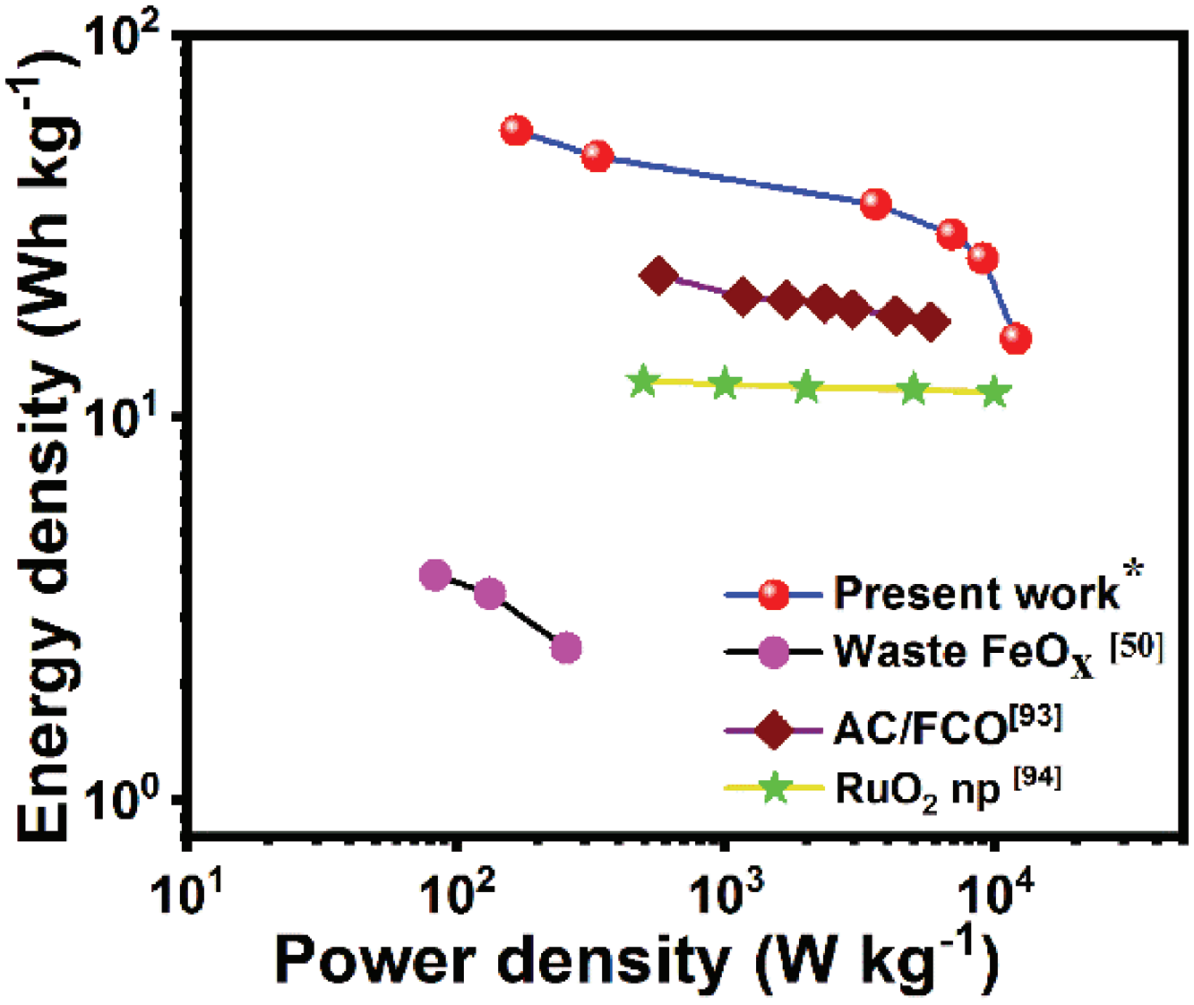
Comapritive Ragone plot showing energy density and power density relationship of a few supercapcitor materials: Industrial Mill Scale Waste (waste FeO*_x_*),^48^ AC/FCO nanocomposites,^93^ ruthenium oxide nanoparticle (RuO_2_ np) in a two‐electrode system,^94^ and RM‐10 sample (present work).

Ragone plots^92^ are often used to express power densities and energy densities of a supercapacitor and represented in **Figure** **9** for the RM‐10 sample. The sp. energy density *E*_s_ (W h kg^−1^) was calculated as^70^

(6) $$E_{s}=0.5C_{s}V^{2}$$

where *C*_s_ is the sp. capacitance obtained from CD and *V* is the effective potential window. The sp. power density *P*_s_ (W kg^−1^) was calculated using the following equation

(7) $$P_{s}=\frac{E_{s}}{t_{d}}$$

where *E*_s_ is the sp. energy density and *t*_d_is the discharge time.

Performance of the RM‐10 electrodes are compared with other metal oxide‐based supercapacitors in terms of energy density (W h kg^−1^) and power density (kW kg ^−1^) in a Ragone plot (Figure 9). The comparative plot shows that the RM‐10 samples exhibit better performance as compared to iron oxide rich industrial mill scale waste (FeO*_x_*),^48^ composite metal oxides activated carbon/iron cobalt oxide (AC/FCO) electrode,^93^ and a two‐electrode supercapacitor comprised of ruthenium oxide nanoparticles.^94^

The highest energy density was measured as 56 Wh kg^−1^ at a sp. current density of 1 A g^−1^ and a maximum power density of 12 kW kg^−1^ at a higher current density of 20 A g^−1^. Even at this higher current density, the sp. energy density was evaluated as ≈16 Wh kg^−1^ indicating a better stability of the electrode even at high current density.

#### 2.4.3 Electrochemical Impedance Spectroscopy (EIS) Study

**Figure 10:**
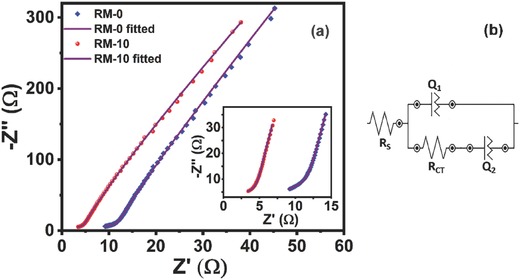
a) Nyquist plot of experimental and fitted impedance data for RM‐0 (blue diamond) and RM‐10 (red dot), the violet line corresponds to fitted data. Inset depicts the zoomed view of impedance data at high‐frequency region. b) corresponds to the model equivalent electrical circuit.

In order to understand the ionic diffusion and charge transfer kinetics of the RM electrodes, EIS has been employed. EIS is a nondestructive, fast, and simple technique to excerpt the electrode kinetics of the test material.^95^ Here the real part of impedance is plotted against the imaginary part (Nyquist plot). In this study, a perturbation voltage with an r.m.s. value of 10 mV was applied while the frequency was varied from 0.1 to 100 kHz. The Nyquist plots of RM‐0 and RM‐10 samples are represented in **Figure** **10**a. The plot can be categorized in two separate regions. A semicircular nature was observed in the high frequency region and it had a protracted tail in lower frequency regimes. The linear tail originates from the frequency‐dependent ion transport and/or diffusion of ions at outer and inner surfaces of the electrode.^96^ The magnitude of the real part of the impedance is slightly decreased after the ball‐milling process (for clarity, the region is magnified in the inset of Figure 10a). The diameter of the semicircle of the Nyquist plot determines the charge transport resistance of a system.^97^ The decrease in the diameter of the RM‐10 sample favors diffusion of the electrolyte in the electrode surface.

The EIS data were further modeled and fitted using a model equivalent electrical circuit (Figure 10b). The equivalent circuit consists of a series resistance, a charge transfer resistance, and two constant phase elements (CPE) as described by Fu et al.^48^ and Bisquert et al.^98^ The series resistance takes care of the cumulative effects of sum of contact resistance, material resistance, and electrolyte resistance.^79^ The CPE (*Z*) is defined as *Z* = *Y*_0_ (*jω*)^−α^, where *Y*_0_ = 1/*C* for α = 1 and, *Y*_0_ = *R* for α = 0; *C* and *R* represents the capacitance and resistance, respectively. α is the exponent of CPE. When α = 0, this represents a purely resistive element whereas α = 1 means the component is purely capacitive in nature.^99^ The first CPE represents the conventional double layer and redox capacitance along with the nonlinearity and the second one represents the CPE at low‐frequency region which appears due to the roughness at the blocking interface,^98^ nonlinearity, and diffusion driven intercalation.

The fitted curves for RM‐0 and RM‐10 samples showed residual χ^2^ values of 0.001 and 0.002 respectively confirming an excellent fitting. The values obtained from the equivalent circuit fitting are also tabulated in **Table** **3**. The charge transfer resistance for RM‐0 was evaluated as 17 Ω, which reduced to 11 Ω in RM‐10 sample. The series resistance was also reduced to 3 from 9 Ω in case of RM‐10 sample. The minimal equivalent series resistance and charge transfer resistance of RM‐10 sample can be related to its smaller size and highly ordered and uniform morphology which contributes to an advantageous intrinsic electronic conductivity.^100^ The minimal charge transfer resistance also indicates a high diffusion of electrolytes. The constant phase element in the equivalent circuit for RM samples also verifies the presence of both capacitive and diffusion governed charge storage (intercalation) process which again proves the complex charge storage mechanism and diffusion dominated pseudocapacitance.

**Table 3:** Different component of fitted parameters obtained from equivalent model electrical circuit fitting from Nyquist plot of impedance spectroscopy

Sample	*R*_S_ [Ω]	*R*_CT_ [Ω]	*Q*_1_ [µmho]	*Q*_2_ [µmho]	χ^2^
RM‐0	9.1	17.3	4.0; 0.9	8.7; 0.8	0.001
RM‐10	3.4	11.2	5.6; 0.9	9.1; 0.8	0.002

## 3 Conclusion

The present work successfully validated the suitability of mechanically activated red mud as a potent source of energy storage material. Ball milling offers uniform morphology and higher charge transfer kinetics, from this industrial waste material. In‐depth electrochemical performance assessment showed that the RM‐10 modified GCE exhibited high sp. capacitance, high energy density, and power density with a remarkable long‐term cyclic stability. The storage mechanism was found to be diffusion‐dominated in nature. The calculated coulombic efficiency also confirmed the battery‐like pseudocapacitance of ball‐milled RM‐10 sample. Importantly, the present approach to producing a high‐performance supercapacitor electrode material from industrial waste is green, inexpensive, and sustainable. The method also deals with an alternative path toward waste management and sustainability. In future, the performance could further be improved by producing hybrid supercapacitors using mechanically treated waste red mud.

## 4 Experimental Section

*Material*: RM was collected from National Aluminum Company Limited (NALCO), India. The as‐received powder was dehydrated for an hour at an elevated temperature (383 K). The powder was further ground in an automated motor‐pestle for 1 h to produce the fine powder. All the electrolytes used herein were purchased from Fisher Scientific. All the aqueous solutions were produced with ultrapure de‐ionized (DI) water (Millipore‐Q systems: electrical resistivity 18 MΩ cm at room temperature (298 K)).

*Method*: In order to prepare the RM nanoparticles, mechanical milling using a planetary ball mill (Retsch, PM200) was employed. The powders were placed in a chrome steel bowl (volume 60 mL) filled with steel balls of diameter 5 mm. The ball‐to‐powder mass ratio of 8:1 (ball: RM) was used. Milling in the present study has been carried out at 150 rpm and was continued up to 15 h with intermediate intervals of 1 h.

*Characterization—Morphological Measurement*: In order to analyze the chemical composition of RM‐0, X‐ray fluorescence microscopy was carried out and the elemental details are confirmed using TEM‐EDX spectroscopy (Bruker S4 PIONEER). The morphology and particle size were monitored using FESEM and TEM (JEOL 2100F). The particle size was measured using a particle size analyzer (Melvin). The crystallinity and the crystal phases were determined by X‐ray diffraction technique using Bruker D8‐Discover with Cu‐*K*_a_ radiation (λ = 0.154 nm) The Raman spectra were recorded using a Renishaw Raman spectrometer (inVia) using a 532 nm Laser source, using nominal power of 25 mW for 60 s, 50× magnification.

*Characterization—Electrochemical Measurement*: The CV, galvanostatic CD, and EIS measurements were performed on an Autolab Potentiostat Galvanostat PGSTAT302N (Metrhom, Netherlands). For the electrochemical characterization, CV measurements were carried out in a three‐electrode setup, consisting of Ag/AgCl as the reference electrode, platinum wire as the counter electrode, and GCE modified with red mud particles as the working electrode. For this purpose, 5 × 10^−3^ m of potassium ferro/ferry cyanide in 0.1 m KCl was used as the standard electrolyte. The CV scans were measured between −0.3 and 0.7 V. In order to find out the sp. capacitance from the CV, a similar set of electrodes were utilized, and the measurements were recorded in a 6 m KOH electrolyte solution within the scan range −1.0 to 0.2 V. The scan rates were varied, and the sp. capacitances were evaluated from the CV curve. The galvanostatic charging/discharging analysis was recorded in a chronopotentiometry mode with the same three‐electrode setup and similar electrolyte (6 m KOH) as used in the CV measurements. For the CD analysis, the predefined cutoff voltages were obtained from the CV measurements. The impedance measurements were carried out in the frequency response analyzer (FRA) potential scan mode with a similar electrode–electrolyte setup where red mud coated GCE acts as the working electrode, along with platinum wire counter and Ag/AgCl reference electrode. All the measurements were carried out at room temperature. The active mass and electrochemically active surface area were calculated from CV data as discussed in our previous reports.^70^, ^99^ For EIS measurements, a sinusoidal alternating current (a.c.) voltage with root mean square (r.m.s.) value of 10 mV was applied as a perturbation. The frequency of the a.c. voltage was varied from 0.1 Hz to 100 kHz. The as‐obtained Nyquist plots were fitted using the vendor provided NOVA (version 1.11) software.

## Conflict of Interest

The authors declare no conflict of interest.

## Supporting information

Supplementary
