# Supplementary material for: Effective Utilization of Waste Red Mud for High Performance Supercapacitor Electrodes
Source: Glob Chall. 2018 Oct 25;3(2):1800066. doi: 10.1002/gch2.201800066 (PMC6607351; doi:10.1002/gch2.201800066)
Supplement: Supplementary file 1 — Supplementary [file GCH2-3-na-s001.pdf]

Copyright WILEY-VCH Verlag GmbH & Co. KGaA, 69469 Weinheim, Germany, 2018.

Global Challenges
Open Access

Supporting Information

for *Global Challenges,* DOI: 10.1002/gch2.201800066

Effective Utilization of Waste Red Mud for High Performance Supercapacitor Electrodes

Gourav Bhattacharya, Sam Jeffery Fishlock, Joy Sankar Roy, Anurag Pritam, Debosmita Banerjee, Sujit Deshmukh, Subhasis Ghosh, James A. McLaughlin, and Susanta Sinha Roy**

Supporting information

Effective Utilization of Waste Red Mud for High Performance Supercapacitor Electrodes

Gourav Bhattacharya[a,b], Sam Jeffery Fishlock[a], Joy Sankar Roy[b], Anurag Pritam[b], Debosmita Banerjee[b], Sujit Deshmukh[b], Subhasis Ghosh[c], James A. McLaughlin[a*], Susanta Sinha Roy[b*]

[a]Nanotechnology and Integrated Bioengineering Centre, University of Ulster, Jordanstown Campus, Newtownabbey, BT37 0QB, Northern Ireland, UK.

[b]Department of Physics, School of Natural Sciences, Shiv Nadar University, Gautam Buddha Nagar 201314, Uttar Pradesh, India.

[c]School of Physical Sciences, Jawaharlal Nehru University, New Delhi 110067, India

E-mail: susanta.roy@snu.edu.in, jad.mclaughlin@ulster.ac.uk

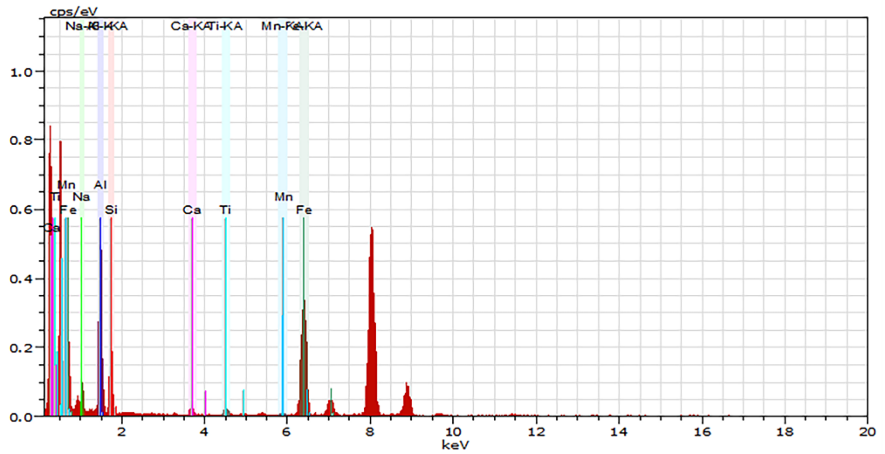

Figure S1. TEM-EDAX image of RM-0.

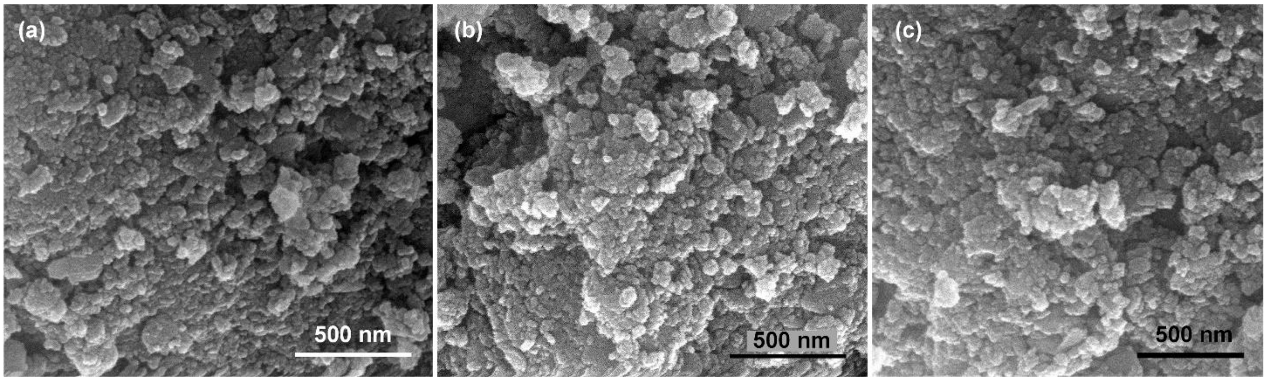

Figure S2. FESEM images of (a) RM-5, (b) RM-10 and (c) RM-15.

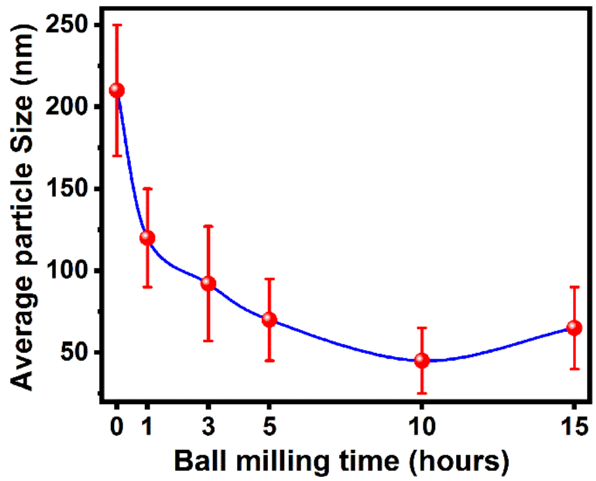

Figure S3. Variation of average particle size of red mud as a function of milling time.

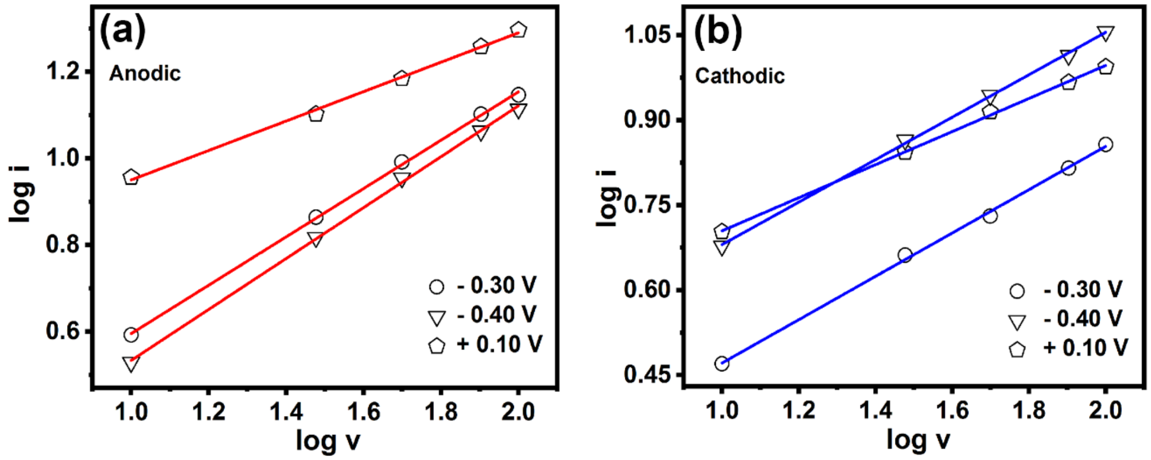

Figure S4. "log i" vs. "log v" plot. Plot showing linear relationship of (a) cathodic (b) anodic sweep of cyclic voltammogram at different potential

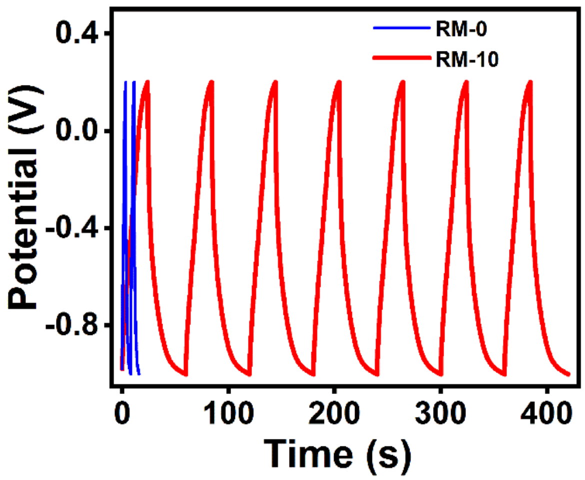

Figure S5. Galvanostatic charge/discharge curve of RM-0 and RM-10 in 6 M KOH solution at sp. current 6 A g^{-1}.

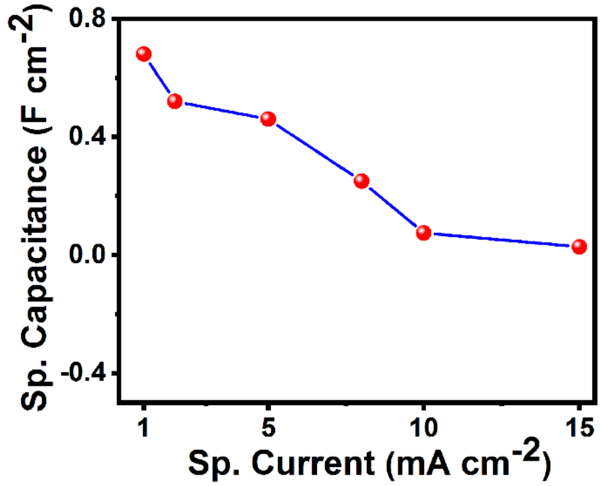

Figure S6. Variation of sp. areal capacitance with specific current for RM-10.
